# Recombinant chymase inhibits fibrinolysis induced by endogenous plasmin in clotted human blood

**DOI:** 10.3389/fimmu.2025.1511990

**Published:** 2025-04-17

**Authors:** Laurence Vincent, Catherine Lapointe, Patrick P. McDonald, Christos Rammos, Tienush Rassaf, Maria Köllnberger, Hanna Tinel, Stefan Heitmeier, Pedro D’Orléans-Juste

**Affiliations:** ^1^ Department of Pharmacology and Physiology, Faculty of Medicine and Health Sciences, Université de Sherbrooke, Sherbrooke, QC, Canada; ^2^ UNC Blood Research Center, University of North Carolina at Chapel Hill, NC, United States; ^3^ Pulmonary Division, Faculty of Medicine and Health Sciences, Université de Sherbrooke, Sherbrooke, QC, Canada; ^4^ Department of Cardiology and Vascular Medicine, West German Heart and Vascular Center, University of Duisburg-Essen, Essen, Germany; ^5^ Bayer AG, Research & Development, Pharmaceuticals, Wuppertal, Germany

**Keywords:** chymase, mast cells, fibrinolysis, plasmin, fulacimstat, thrombolysis assay, human blood clots, human pulmonary emboli biopsies

## Abstract

**Background:**

Our group recently reported that chymase, a serine protease synthetized and released by mast cells, plays a pivotal role in thrombi stabilization in murine deep vein thrombosis (DVT) models, by interfering with the fibrinolytic properties of endogenous plasmin within thrombi. However, the impact of mast cell-derived chymase on endogenous plasmin activity in human blood clots has yet to be explored.

**Methods:**

The antifibrinolytic properties of human recombinant chymase (rCMA-1) were investigated using an *in vitro* thrombolysis assay based on halo-shaped human blood clots. In addition, a fluorogenic assay was used to detect chymase activity in human thromboembolic biopsies. In both assays, the activity of human chymase was validated using a specific chymase inhibitor, fulacimstat (BAY 1142524).

**Results:**

Although rapidly neutralized in plasma, rCMA-1 remains active within the local microenvironment of a blood clot, inducing resistance to endogenous plasmin-mediated fibrinolysis in the presence of recombinant tissue plasminogen activator (tPA). This leads to a concentration-dependent decrease in clot lysis by rCMA-1. The plasmin-inactivating properties of rCMA-1 were inhibited by fulacimstat, resulting in an accelerated clot dissolution. The pro-fibrinolytic effects of fulacimstat in human blood clots were reversed by a plasminogen/plasmin inhibitor, BAY 1214237. Finally, fulacimstat-sensitive chymase activity was identified in thrombi collected from thrombectomy patients, supporting the potential role of the mast cell-derived serine protease in human blood clot stabilization under pathological conditions.

**Conclusion:**

These *in vitro* experiments with human whole blood suggest that mast cell-derived chymase impairs blood clot resolution by interfering with the fibrinolytic activity of endogenous intra-clot plasmin. Our findings provide evidence that recombinant mast cell chymase interferes with endogenous plasmin activity in human whole blood clots *in vitro* and support the potential of chymase inhibitors, such as fulacimstat, as fibrinolytic agents for thrombotic and thromboembolic disorders.

## 1 Introduction

Arterial and venous thrombi are major contributors to the global burden of disease with significant morbidity and mortality (1). The current pharmacotherapy for venous thromboembolism (VTE) focuses on the prevention of fibrin formation using anticoagulants such as heparins, vitamin K antagonists (VKAs), and direct oral anticoagulants (DOACs) (2–4). While pharmacotherapy for arterial thrombosis relies heavily on rapid thrombolysis with agents like recombinant tissue plasminogen activator (tPA), these treatments carry a significant risk of bleeding (5). Although DOACs are effective in preventing thrombosis and have a lower bleeding risk, they are less efficient in dissolving existing clots (6). This underscores the need for new antithrombotic agents that can both prevent and treat thrombosis with minimal impact on hemostasis.

Mast cells are immune cells that reside in various tissues, playing a pivotal role in innate inflammatory responses and allergic reactions. Recent studies have suggested that mast cells granular contents are crucial in the development of deep vein thrombosis (DVT) in the mouse model (7–9). Upon activation, mast cells release cytoplasmic granules containing several preformed inflammatory mediators, such as histamine, heparin, cytokines, and mast-cell proteases like chymases, tryptases, cathepsin G, and carboxypeptidase A3 (10). Mast cell-derived chymase is involved in the hydrolysis of many physiological substrates, including angiotensin I (11), big endothelin-1 (12), and extracellular matrix proteins like fibronectin, procollagenase and pro-matrix metalloproteinase (pro-MMP)9 and 2 (13, 14), contributing to tissue remodeling, inflammation, and vascular function. In humans, only one α-chymase (CMA-1, human chymase) is expressed (14).

In a previous study, our group demonstrated that mast cell-derived chymase plays a major role in thrombus stabilization in mouse models (15). In the latter study, both genetic and pharmacological inhibition of chymase effectively prevented and resolved thrombus formation in a mouse model of DVT, without increasing bleeding time. The inhibitory properties of recombinant mouse or human chymase on purified plasmin was also demonstrated *in vitro*. Finally, chymase-containing mast cells were identified in human DVT biopsies. However, whether chymase impairs endogenous plasmin’s fibrinolytic properties in human thrombi remains unexplored.

Bonnard et al. (16) introduced a novel high-throughput thrombolysis assay using citrated whole blood samples to form halo-shaped clots in 96-well plates, offering several advantages over currently available methods such as rotational thromboelastometry (ROTEM) (17). Notably, this high-throughput assay requires only 20 µL of whole blood. Supplementation of calcium chloride (CaCl_2_) to citrated whole blood initiates clotting through recalcification (18, 19), resulting in halo-shaped blood clots with an empty center. This methodological approach allows for precise monitoring of the thrombolytic process over time as the release of red blood cells leads to an increase in absorbance (16).

In the present study, we investigated the antifibrinolytic properties of recombinant human chymase on endogenous plasmin in human blood clots. Although chymase is rapidly inactivated in blood by endogenous circulating inhibitors, including alpha 1-antitrypsin, alpha 1-antichymotrypsin and alpha 2-macroglobulin (20, 21), our laboratory has recently reported that chymase contained within thrombi significantly reduced plasmin activity in the murine model (15). These latter results support the potential of chymase inhibitors as fibrinolytic agents.

The present study thus aimed to validate the plasmin-inactivating properties of chymase as well as the fibrinolytic properties of an inhibitor of the mast cell-derived protease in clotted human blood.

The chymase inhibitor fulacimstat (BAY 1142524) has been proven safe and well-tolerated in clinical trials (22–25). Furthermore, the selectivity of fulacimstat has been tested against various serine proteases of the hemostatic system, including plasmin, plasma kallikrein, thrombin, tissue plasminogen activator, factor Xa, and factor XIa, demonstrating a half maximal inhibitory concentration (IC_50_) above 2 µM compared to 4 nM for chymase. To identify specific pathways of fibrinolysis inactivation, the present study also employed BAY 1214237, a plasminogen/plasmin inhibitor with an IC_50_ of 4.43 μM (15).

The present study suggests that chymase inhibits, in a fulacimstat-sensitive fashion, the fibrinolytic properties of endogenous plasmin, thereby impeding the effective resolution of clotted human blood.

## 2 Methods

### 2.1 Recombinant enzymes, drugs, and chemicals

Activated recombinant CMA-1 (rCMA-1), the chymase inhibitor fulacimstat (BAY 1142524, 1-(3-methyl-2-oxo-1,3-benzoxazol-6-yl)-2,4-dioxo-3-[(1R)-4-(trifluoromethyl)-2,3-dihydro-1H-inden-1-yl] pyrimidine-5-carboxylic acid), the plasmin/plasminogen inhibitor BAY 1214237, (10‐chloro‐2‐oxo‐1,2‐dihydropyrimido[1,2‐b] indazol‐4‐yl) piperidinium chloride, rivaroxaban and the fluorogenic substrate for chymase activity, Abz-HPFHL-Lys(Dnp)-NH_2_, were obtained from Bayer AG (Wuppertal, Germany). Human purified plasmin was purchased from Innovative Research (Novi, MI, USA).

### 2.2 Blood collection

Blood was collected from healthy donors under a protocol approved by the Comité institutionnel d’éthique de la recherche (CIER) of the Université de Sherbrooke. All subjects gave written informed consent in accordance with the Declaration of Helsinki. Whole blood was collected via venipuncture into sodium citrate (3.2% w/v final), and stored at 4°C for up to 7 days.

### 2.3 Clot formation and extraction of chymase and plasmin

Blood clots (50 µl), in the presence or absence of rCMA-1 (1 µM), were generated by adding 31.2 mM of calcium chloride (CaCl_2_) to blood samples and incubated at 37°C for 30 min to allow solidification. In another series of experiments towards the development of a reliable biomarker assay, fulacimstat (final concentration of 1 to 100 µM) was added to blood samples. For chymase extraction, clots were homogenized in 10 volumes (v/v) of 20 mM PBS (pH 7.4) supplemented with 0.5 mg/mL of BSA using a glass‐Teflon homogenizer (15), then transferred to a 1.5 ml micro-tube. The homogenates were incubated at 37°C for 30 min, and centrifuged at 18,000x*g* at 4°C for 30 min, and the supernatants were kept at 4°C until tested for chymase enzymatic activity. Plasmin was purified from clots as previously described (15). Clots were homogenized in 10 volumes (v/v) of 20 mM PBS (pH 7.4) supplemented with 1.5 mg/mL of BSA using a glass‐Teflon homogenizer, then transferred to 1 mL ultracentrifuge tubes. The homogenates were incubated at 37°C for 30 min, and centrifuged at 100,000xg at 4°C for 20 min. Supernatants were kept at 4°C until tested for plasmin enzymatic activity.

### 2.4 Measurement of plasmin and chymase enzymatic activity in recalcified whole blood clots

A volume of 30 μl of supernatants from clot extracts was treated with either vehicle (0.1% DMSO) or fulacimstat (10 μM) in a white 96‐well plate. In another series of experiments, the plasmin/plasminogen inhibitor, BAY 1214237 (concentration ranging from 0.1 to 50 μM), with blood clots extracts or purified plasmin (1 nM) were added to the plate. A total of 65 to 70 μl of 20 mM PBS (pH 7.4) supplemented with 1.5 mg/ml of BSA (for plasmin) or 0.5 mg/ml of BSA (for chymase) was added to each well for a final volume of 100 μl. Plasmin activity was determined by the hydrolysis rate of 50 μM of the fluorogenic substrate, H-D-Ala-Leu-Lys-7-amino-4-methylcoumarin (Bachem AG, Bubendorf, Switzerland) at 37°C for 1 hour. The fluorescence of the released 7-amino-4-methylcoumarin (AMC) molecule was measured in duplicate with an Infinite M1000 spectrophotometer (Tecan Group Ltd., Männedorf, Switzerland) with an excitation wavelength (λ_ex_) of 370 nm and emission wavelength (λ_em_) of 460 nm. Chymase activity was determined by the hydrolysis rate of 10 μM of the fluorogenic substrate, Abz-HPFHL-Lys(Dnp)-NH_2_, at 37°C for 1 hour with an λ_ex_ of 320 nm and λ_em_ of 400 nm. Increases in fluorescence are expressed as relative fluorescence intensity in arbitrary units per minute (AU/min^-1^).

### 2.5 Formation of halo-shaped blood clot

The formation of halo-shaped blood clots was conducted following the methods described by Bonnard et al. (16). To initiate clot formation, 31.2 mM of CaCl_2_ was added to blood samples collected from healthy donors. A volume of 20 µl of the CaCl_2_-supplemented blood was then carefully added in a circular motion around the edge of wells in a flat-bottomed, transparent 96-well plate, creating a halo-shaped clot through fluidic cohesion, leaving the center of each well clear. The plate was subsequently sealed and incubated at 37°C for 30 minutes to allow the halos to solidify. Initial control experiments showed that rCMA-1 (1 µM) *per se* did not alter clot stability (results not shown).

### 2.6 Halo-shaped blood clots with subsequently added human chymase

Following the formation and solidification of the halo-shaped blood clots as described above, fully formed clots were simultaneously treated with recombinant human tissue plasminogen activator (tPA; 1.5 nM; Cathflo^®^, Genentech Inc., SF, USA) and recombinant human chymase (at a final concentration of 0.5 µM or 1 µM) in phosphate-buffered saline (PBS), in the presence or absence of fulacimstat at concentrations of 0.1, 1, or 10 µM (only applied at 1 µM rCMA-1). In another set of experiments, BAY 1214237 (1 µM) was co-administered with tPA, recombinant chymase (1 µM), and fulacimstat (10 µM) onto the pre-formed halos. The plate was sealed and incubated for 2 hours at 37°C with gentle lateral mixing (2 rpm). Absorbance at 510 nm was then measured, and clot degradation was calculated using the formula:


$$D_{x}\left(t\right)=100\times \frac{A_{x}\left(t\right)-A_{zero}\left(t\right)}{A_{total}\left(t\right)-A_{zero}\left(t\right)}$$

### 2.7 Formation of halo-shaped blood clot containing human chymase

In a different set of experiments, recombinant human chymase (rCMA-1) was incorporated during the initial formation of the halo-shaped blood clots. A volume of 1 µl of activated rCMA-1 was introduced to achieve final concentrations of 0.5 µM or 1 µM, in the presence or absence of 10 µM fulacimstat (used only at the 1 µM concentration of rCMA-1). The chymase was deposited around the bottom edge of the wells, and 20 µl of CaCl_2_-supplemented blood was then added in a circular motion to facilitate the halo formation. The plate was sealed and incubated at 37°C for 30 minutes to allow the blood halos to solidify.

### 2.8 Thrombolysis assay and analysis of fibrinolytic kinetics

Recombinant tPA (Cathflo^®^, Genentech Inc., SF, USA) induces blood clot fibrinolysis by activating endogenous plasmin. tPA was diluted to a concentration of 1.5 nM in 80 µl of 10 mM PBS (pH 7.4) and was added into each well. The 96-wells plate was subsequently incubated at 37°C for 30 min. Negative controls (A_total_) were obtained from addition of 80 µl of PBS without fibrinolytic agents, and positive controls (A_zero_) correspond to wells containing 20 µl of fresh whole blood, non-supplemented with CaCl_2_, and 80 μl of 10 mM PBS (pH 7.4). The fibrinolysis rate was measured with a plate reader (Infinite M1000 spectrophotometer (Tecan Group Ltd., Männerdorf, Switzerland)). The degradation of the halo was measured in real time as coverage of each experimental well as the thrombolysed blood increases absorbance at 510 nm. Changes in absorbance were measured every minute at 37°C for 2h, after a 5 second orbital shaking at 195.3 rpm at an amplitude of 3.5 mm. Maximal absorbance measurement was converted to a percentage of degradation according to the formula above mentioned.

In addition to real time monitoring and plotting of blood degradation kinetics, other parameters derived from the same curves, namely: i) The activation time (A_t_) required for the induction of fibrinolysis which is defined as the first value of t for which D_x_(t)/dt > 1. ii) The time required to obtain 50% lysis (T0.5) is defined as the first value of *t* which D_x_(t) = 50%. iii). The maximum clot lysis rate (CLR_max_) corresponds to the maximum value of D_x_(t)/dt.

### 2.9 Measurement of chymase activity in human *ex vivo* thrombi

Fresh thrombi obtained from thrombectomy patients undergoing pulmonary embolectomy for intermediate-high risk pulmonary embolism were immediately cooled and transported to the laboratory for processing on the same day. Each thrombus was meticulously dissected into distinct “whitish” and “reddish” sections, each weighing approximately 50 mg. These sections were incubated in 0.5 mL of PBS and stored at 4°C for 18-48 hours to allow the transition of hemoglobin into the buffer. The samples underwent two washes with 0.5 mL PBS and were gently dried. Subsequently, each sample was further subdivided into smaller pieces of around 8 mg. Each 8 mg thrombus piece was then transferred to a 96-well microtiter plate, and 130 µL of PBS was added. A volume of 15 µL of PBS with either vehicle or fulacimstat (1 µM) was added to each well. Chymase activity was determined by the hydrolysis rate of 10 μM of the fluorogenic substrate, Abz-HPFHL-Lys(Dnp)-NH_2_, at 37°C for 30 minutes with an λ_ex_ of 320 nm and λ_em_ of 420 nm. Increases in fluorescence are expressed as fluorescence intensity in arbitrary units.

### 2.10 Statistical analysis

All data are presented as mean ± standard error of the mean (SEM). All graphical and statistical analysis were performed on GraphPad Prism 10.4.1 software (GraphPad Software, La Jolla, CA). Normality of data distribution was assessed with the D’Agostino & Pearson test for large samples and the Shapiro-Wilk test for smaller samples. Student’s *t*-test was used for two-groups comparisons, as the data met the normality assumption. One-way ANOVA followed by Bonferroni’s *post hoc* test was applied for comparisons between more than tho groups. Two-way ANOVA with Fisher’s LSD test was used for experiments involving two factors. Statistical significance was reached when the *P* value was < 0.05, and one symbol (*) represents *P* < 0.05, two symbols (**) represent that *P* < 0.01, and three symbols (***) represent that *P* < 0.001.

## 3 Results

### 3.1 BAY 1214237 inhibits purified or endogenous plasmin enzymatic activity

**Figure 1:**
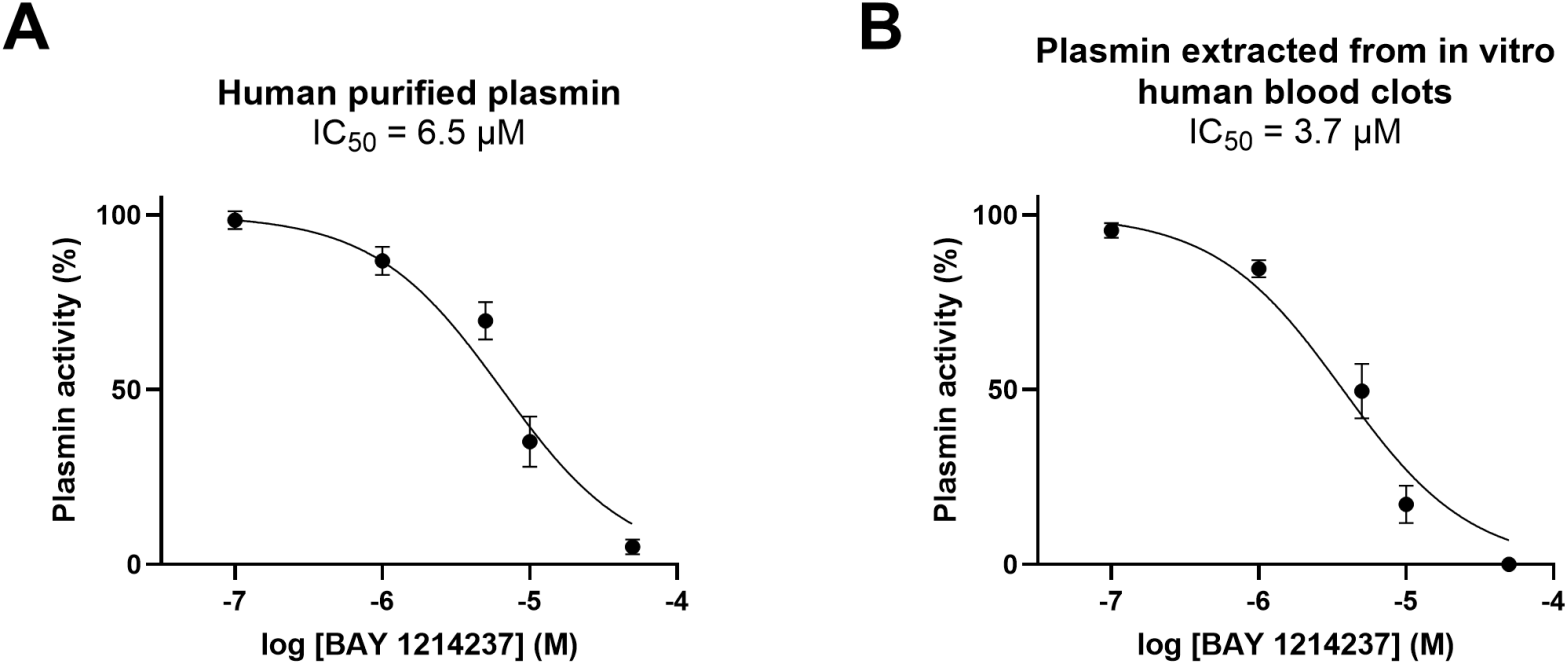
Effect of the plasminogen/plasmin inhibitor, BAY 1214237, on plasmin enzymatic activity. The effect of increasing concentrations of BAY 1214237 on the hydrolytic activity of **(A)** purified human plasmin (n = 4-8) and **(B)** endogenous plasmin extracted from blood clots (n = 6-10) was assessed. Plasmin activity was measured as the 7‐amino‐4‐methylcoumarin (AMC)–specific cleavage (nM) of the fluorogenic substrate H-D‐Ala‐Leu‐Lys‐AMC. Results are presented as mean ± SEM.

In a first series of experiments, the plasminogen/plasmin inhibitor BAY 1214237, inhibited, in concentration-dependent fashion, purified- as well as endogenous plasmin extracted from clots formed *in vitro* using human whole blood, with a half maximal inhibitory concentration (IC_50_) of 6.488 and 3.718 µM, respectively (**Figures 1A, B**).

### 3.2 BAY 1214237 inhibits plasmin-mediated fibrinolysis of human halo-shaped blood clots

**Figure 2:**
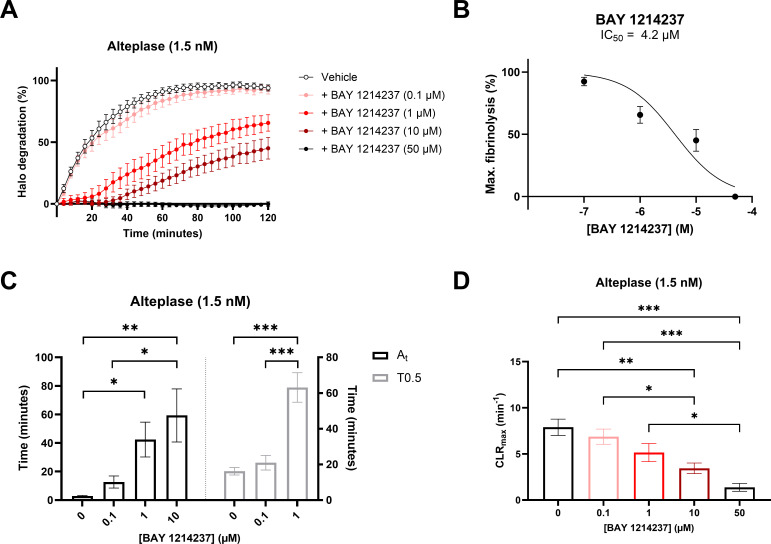
Pharmacological characterization of a plasminogen/plasmin inhibitor, BAY 1214237, on human halo-shaped blood clot fibrinolysis. **(A)** Thrombolytic profiles of alteplase on human blood clots were measured in the presence of vehicle or increasing concentrations of BAY 1214237 (0, 0.1, 1, 10 and 50 μM). **(B)** Impact of BAY 1214237 on maximal blood clot fibrinolysis. **(C)** Fibrinolysis activation times (A_t_, grey spots), time to 50% lysis (T0.5, black spots) and **(D)** maximal clot lysis rates (CLR_max_) were presented for each concentration. Each point and bar represent the mean ± SEM (n = 8-10). *p < 0.05, **p < 0.01, ***p < 0.001 determined by one-way ANOVA with Bonferroni’s multiple comparisons test.

Complementary to the inhibitory properties of BAY 1214237 on endogenous plasmin activity shown in **Figure 1B**, the same inhibitor demonstrated a concentration dependant reduction in the dissolution of halo shaped-blood clots in the presence of 1.5 nM of Alteplase (**Figures 2A**). Furthermore, increasing concentrations of the plasminogen/plasmin inhibitor BAY 1214237 significantly extended activation times and time to 50% lysis (T0.5), while reducing maximal clot lysis rates (**Figures 2C, D**). Additional control experiments investigated the roles of endogenous Factor Xa and plasmin in the induction of coagulation of halo-shaped human blood clots with increasing concentration of the Factor Xa inhibitor rivaroxaban, the chymase inhibitor fulacimstat or the plasminogen/plasmin inhibitor BAY 1214237 (from 0.01 to 100 µM) (**Supplementary Figure S1**). Rivaroxaban inhibited halo coagulation process with a half maximal inhibitory concentration of 1.249 μM. This effect was not observed with fulacimstat or BAY 1214237, indicating that the latter compounds do not interfere with any coagulation factors. Taken together, these results demonstrate that the halo assay requires the activation of endogenous Factor Xa and plasmin for the induction and dissolution, respectively, of halo-shaped human blood clots.

### 3.3 Exogenous human chymase reduces endogenous plasmin activity in human blood clots

**Figure 3:**
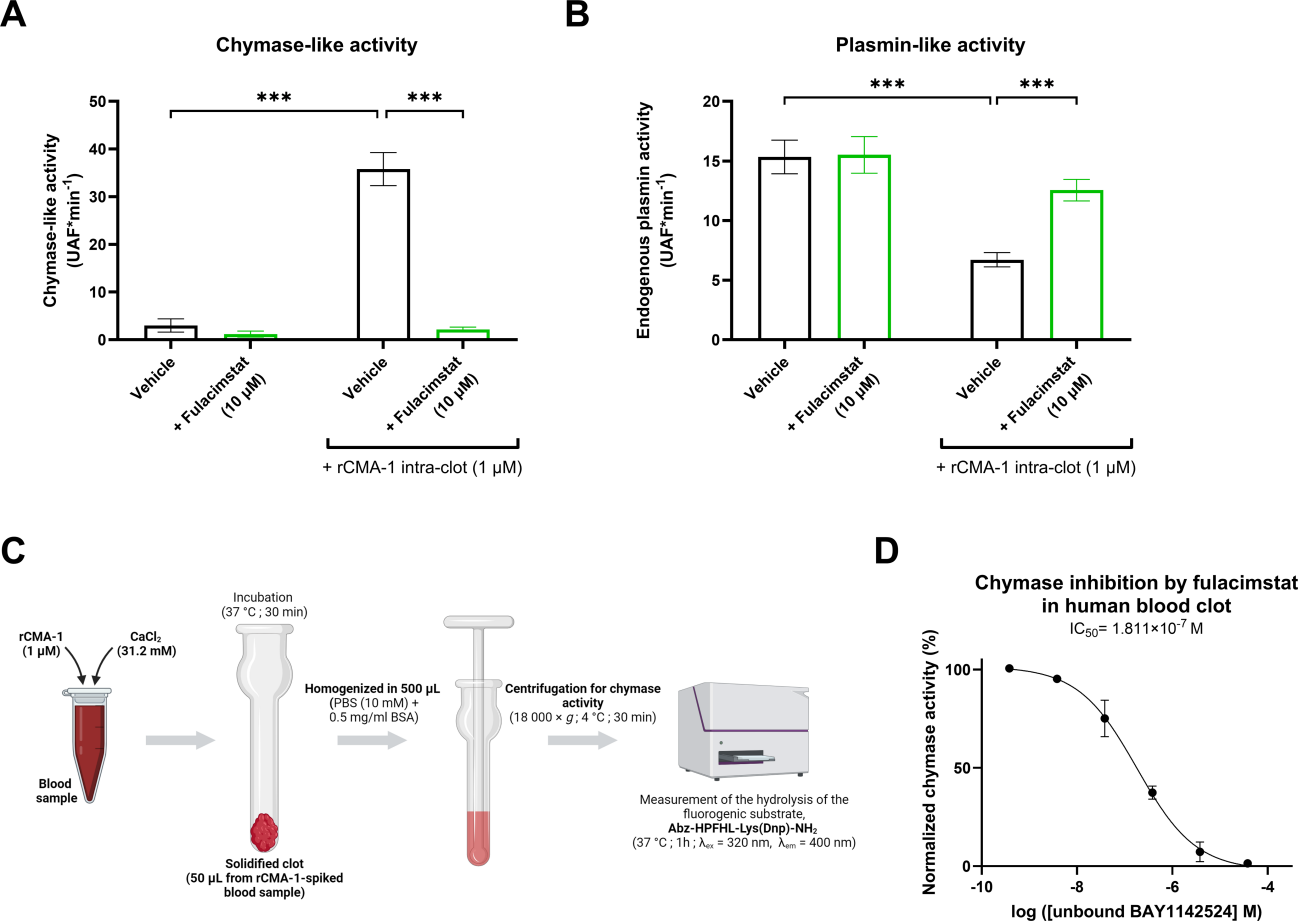
Recombinant human chymase (rCMA-1) reduces endogenous plasmin activity within human blood clots. **(A)** Chymotrypsin-like activity was measured from human blood clots in the presence or absence of exogenous recombinant human chymase (rCMA-1; 1 μM), treated with vehicle or Fulacimstat (10 μM). **(B)** Plasmin-like activity was measured in human blood clots under the same conditions. Plasmin and chymase activity were measured as the 7‐amino‐4‐methylcoumarin (AMC)–specific cleavage (nM) of the fluorogenic substrates H-D‐Ala‐Leu‐Lys‐AMC and Abz-HPFHL-Lys(Dnp)-NH_2_, respectively. **(C)** Schematic representation of a novel biomarker assay for monitoring fulacimstat efficacy in human blood. **(D)** Dose-dependent inhibition of exogenous chymase activity in human blood clots by unbound fulacimstat. Each point and bar represent the mean ± SEM (n = 4-8). ***p < 0.001 determined by two-way ANOVA with Fisher’s LSD test.

The chymase inhibitor, fulacimstat (10 μM), exhibited no effect on endogenous chymotrypsin enzymatic activity in whole blood (**Figure 3A**), yet markedly reduced the hydrolytic activity of exogenously administered human recombinant chymase (rCMA-1: 1 µM), extracted from clots formed *in vitro* using human whole blood. Fulacimstat also demonstrated no effect on plasmin activity in the absence of rCMA-1. In contrast, fulacimstat inhibited the marked reduction of plasmin activity caused by the addition of exogenous rCMA-1 before blood recalcification (**Figure 3B**). In addition, this study showed that fulacimstat inhibited the rCMA-1-dependent degradation of purified human plasmin (**Supplementary Figure S2**) as previously reported with another chymase inhibitor, TY-51469, by our group (15).

In a next series of experiments, a new assay was developed as a biomarker of fulacimstat activity in human blood clots. Using this assay, it was suggested that plasma free concentrations of fulacimstat may be determined in coagulated blood samples of human subjects, as illustrated in **Figures 3C, D**.

### 3.4 Pro-fibrinolytic properties of fulacimstat in human blood clots are BAY 1214237-sensitive

**Figure 4:**
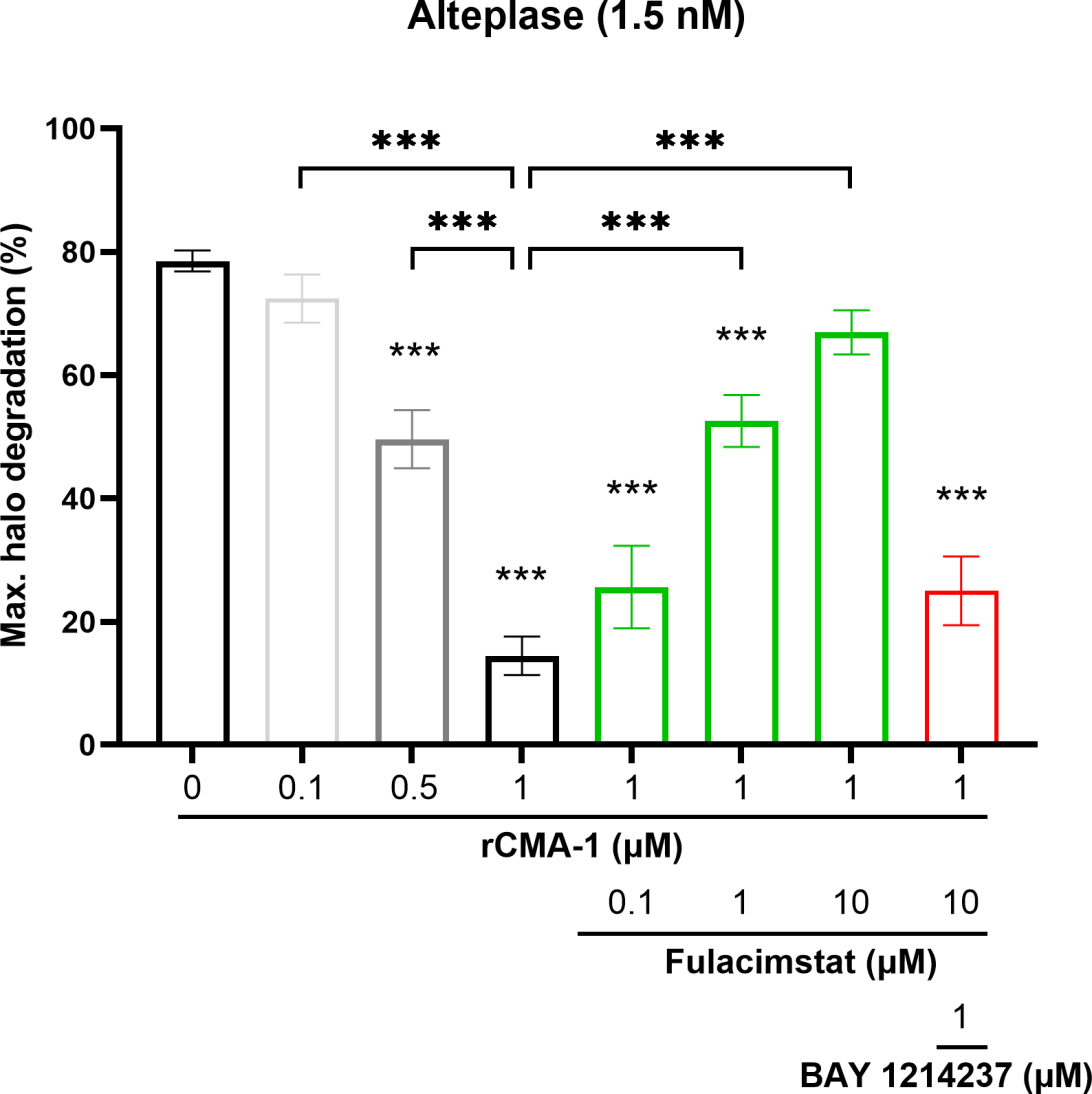
Inhibition of halo-shaped blood clot fibrinolysis by exogenous human recombinant chymase (rCMA-1). Maximal fibrinolysis of a human blood clot by recombinant tPA after 120 minutes, in the presence or absence of increasing concentrations of rCMA-1 (0.1, 0.5 and 1 μM), with or without increasing concentrations of fulacimstat (0.1, 1 and 10 μM), or a combination of the plasminogen/plasmin inhibitor, BAY 1214237 (1 μM) and fulacimstat (10 μM) for the higher concentration of rCMA-1. Each group represents the mean ± SEM (n = 6-13). Statistical significance was determined by one-way ANOVA with Bonferroni’s multiple comparisons test. ***p < 0.001 compared with the vehicle group (asterisks placed directly over each column); comparisons were also made between conditions. Max. indicates maximum.

Considering the significant inhibition of endogenous plasmin hydrolytic activity extracted from blood clot containing rCMA-1, as shown in **Figure 3**, the effects of the addition of the latter serine protease on halo fibrinolysis were investigated using the halo assay. rCMA-1 was administered in the presence of a pre-solidified human blood halos. At the highest concentration tested (1 µM), the human recombinant chymase reduced the tPA-induced dissolution of clots by 80% after 2h incubation (**Figure 4**). The chymase inhibitor (concentrations ranging from 0.1 to 10 µM), administered concomitantly with rCMA-1, restored the tPA-induced fibrinolysis of human blood halos, in a concentration-dependent fashion. Finally, the plasmin/plasminogen inhibitor BAY 1214237 (1 µM), added simultaneously with rCMA-1 (1 µM) and fulacimstat (10 µM), fully reversed the tPA-induced dissolution of human blood halos, as well as the acceleration of halo dissolution in the presence of the chymase inhibitor (**Figure 4**). Taken together, these results showed that exogenous chymase impaired clot dissolution by plasmin, which was reversed by the addition of fulacimstat in the micro-environment of a pre-formed blood clot.

### 3.5 Recombinant human chymase interferes with halo-shaped blood clots fibrinolysis

**Figure 5:**
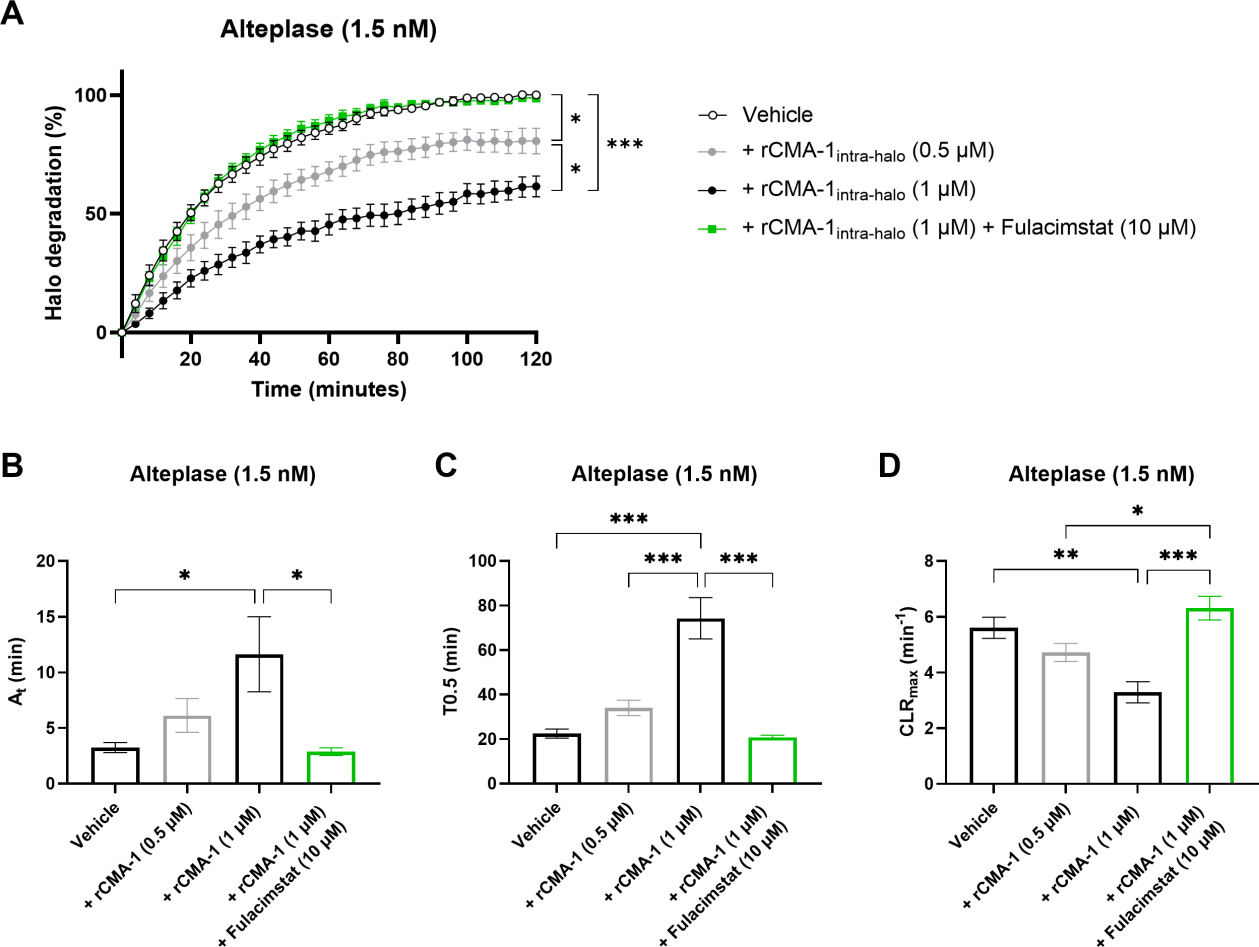
Impaired fibrinolysis profile in halo-shaped blood clots containing human recombinant chymase (rCMA-1). **(A)** Thrombolytic profiles of alteplase on human blood clots containing human recombinant chymase (rCMA-1; 0.5 or 1 μM) were measured in the presence of vehicle or fulacimstat (10 μM). **(B)** Fibrinolysis activation times (A_t_), **(C)** time to 50% lysis (T0.5), and **(D)** maximal clot lysis rates (CLR_max_) were presented for each condition. Each point and bar represent the mean ± SEM (n = 8-10). *p < 0.05, **p < 0.01, ***p < 0.001 determined by one-way ANOVA with Bonferroni’s multiple comparisons test.

Next, recombinant tPA (1.5 nM) induced a marked dissolution of human halo shaped blood clots (**Figure 5A**). This halo dissolution was significantly reduced in concentration-dependent fashion by rCMA-1 when added to the blood prior to CaCl_2_-induced clotting. In addition, the higher concentration of rCMA-1 (1 µM) significantly reduced activation times (A_t_) (**Figure 5B**) and time to 50% lysis (T0.5) (**Figure 5C**), while increasing the maximal clot lysis rates (CLR_max_) (**Figure 5D**). All human chymase-dependant effects were sensitive to fulacimstat.

### 3.6 Fulacimstat-sensible chymase activity identified in human thrombi

**Figure 6:**
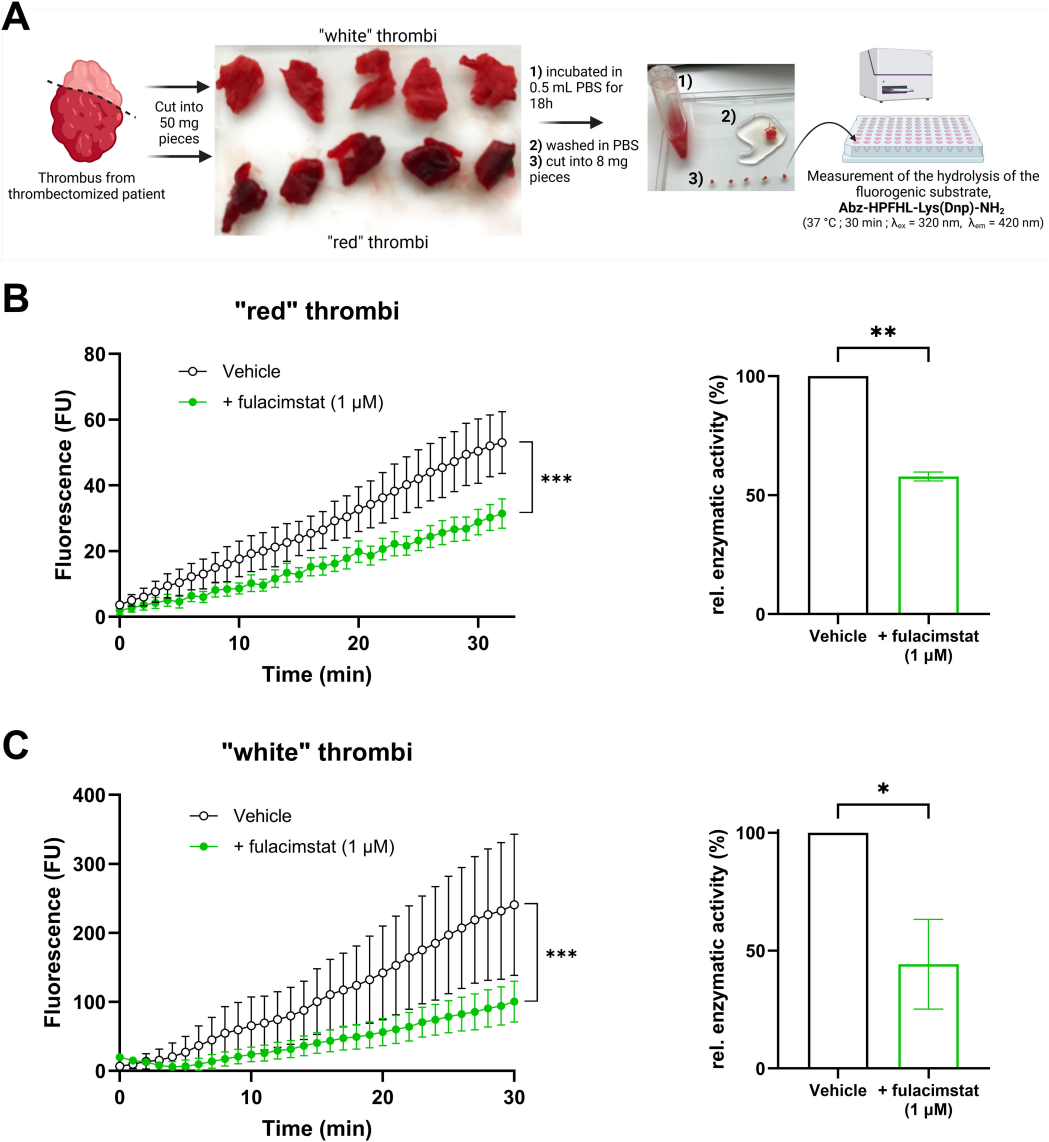
Chymase enzymatic activity in human thrombi *ex vivo*. **(A)** Schematic representation of the preparation of human thrombi samples and the measurement of chymase enzymatic activity using a fluorogenic substrate. **(B)** Fluorescence kinetics of reddish thrombus sections in the presence or absence of fulacimstat (1 µM; left panel) and the corresponding relative enzymatic activity (right panel). **(C)** Fluorescence kinetics of whitish thrombus sections in the presence or absence of fulacimstat (1 µM; left panel) and the corresponding relative enzymatic activity (right panel). Each point and bar represent the mean ± SEM (n = 4-5). *p < 0.05, **p < 0.01, ***p < 0.001 determined by unpaired Student’s *t* test.

In the last series of experiments, chymase activity was identified in human thrombi obtained from thrombectomy procedures (**Figure 6**). All clot samples derived from pulmonary embolectomy exhibited detectable chymase activity, which was sensitive to the specific inhibitor fulacimstat. Notably, the whitish sections of thrombi, typically rich in platelets and fibrin, demonstrated a higher chymase enzymatic activity compared to the reddish sections, which are predominantly composed of red blood cells. These findings represent the first direct evidence of active chymase in human thrombi.

## 4 Discussion

Current pharmacological interventions for rapid thrombus resolution are limited to the use of direct activators of plasminogen to plasmin. However, this particular class of medications is hampered by significant risk of bleeding in patients (26, 27), thus requiring rigorous clinical monitoring (28), and highlighting the need for safer fibrinolytic agents. The present study with chymase in human thrombi suggests that the recently identified antifibrinolytic properties of chymase and profibrinolytic properties of its inhibitor observed in animal experiments could be translatable to the human settings.

Chymase, a serine protease stored in mast cells, is released during mast cell degranulation. Perivascular mast cells are activated for instance under ischemic conditions and release their contents partly into the vascular system. When exposed to whole blood, human chymase is rapidly inactivated by binding to endogenous inhibitors like alpha 2-macroglobulins (20). However, chymase has been shown to remain active in clots during thrombus formation, where it is protected from their circulating inhibitors, as recently reported in animal studies (15). Chymase within the clot can thus contribute to reduced clot lysis by degrading plasmin (15).

Several lines of *in vitro* experiments were conducted in this study to investigate the impact of human chymase on human clots. The perivascular origin and rapid inhibition of chymase in whole blood make direct *in vitro* evaluation in normal hematological tests impossible. Therefore, in this study, assays were developed, which included the *in situ* addition of human chymase to forming clots. When added shortly before or early during clot initiation through recalcification, human chymase remained isolated within the blood clot from its endogenous inhibitors and could exert its enzymatic properties leading to reduced plasmin function. This was demonstrated by an increase in chymase activity and a reduction in plasmin-mediated hydrolysis in the buffer supernatant of the blood clot homogenates. These findings suggest that, upon initiation of coagulation, human chymase is able to reduce the fibrinolytic effects of plasmin within the thrombus, even in the presence of high concentrations of alpha 2-macroglobulins (20).

As described by Bonnard and colleagues (2017), the human halo-shaped blood clot assay is conducted as static model of blood coagulation and fibrinolysis. Our results showed that fulacimstat, conversely to rivaroxaban, does not interfere with human blood coagulation without addition of chymase (**Supplementary Figure S1**). Additionally, tPA-induced fibrinolysis in this model was confirmed to be plasmin-dependent, as evidenced by the inhibitory properties of the specific plasmin inhibitor, BAY 1214237, on both purified and clot-extracted plasmin (**Figure 1**), as well as in tPA-induced clot lysis (**Figure 2**). BAY 1214237 has also been recently used to reverse the antithrombotic properties of the 2^nd^ generation chymase inhibitor TY-51469 (29) in a mouse DVT model (15).

Importantly, neither of the two human blood clot models analyzed in the present study contained endogenous chymase, and thus both required supplementation of the exogenous mast cell-derived serine protease (**Figure 3A**).

On the other hand, the development of a novel biomarker assay to quantify the inhibitory properties of fulacimstat in clotted blood may represents a useful method for monitoring the impact of the chymase inhibitor in future clinical settings. Indeed, the assay described in the present study, may facilitate detection and quantification of plasma-free concentrations of fulacimstat in coagulated blood samples from patients, thus providing a reliable tool for assessing the inhibitor’s efficacy in reducing chymase activity.

Our group has also recently shown the presence of immunoreactive human chymase in DVT derived from patients and have also reported that recombinant CMA-1 inactivates purified human plasmin’s hydrolytic properties in a chymase inhibitor-sensitive manner (15). In the present study, we have demonstrated for the first time the presence of a fulacimstat sensitive-chymase activity in human thrombi obtained from thrombectomized pulmonary embolism patients (**Figure 6**). Thus, albeit further investigation is required, it is suggested that the orally available chymase inhibitor may reduce pathological chymase in human thrombi with no impact in hemostasis.

While the *in vitro* halo assay described by Bonnard and colleagues offers a controlled and reproducible method for studying the fibrinolytic system, the absence of adventitial matrix, ischemic and inflammatory processes, and thrombus neovascularization may limit its ability to mimic the *in vivo* pathological mechanisms associated with the formation and resolution of an actual thrombus. Worthy of notice however, Ponomaryov et al. (7) showed that mast cell depletion abolished venous thrombosis in mice with intact vascular integrity, indicating that a localized perivascular inflammatory environment may be required for mast cell-dependent thrombosis in the vasculature. On the other hand, the halo *in vitro* model used in the present study lacks intrathrombotic neovascularization, an important process that contributes to thrombus resolution by forming new vascular channels within the thrombus (30). As mast cells accumulate at the periphery of venous thrombi (31) and degranulate in response to inflammation, chymase inhibition by fulacimstat could promote fibrinolysis and accelerate thrombus resolution in the context of neovascularization.

It is worth noting that the serine protease cathepsin G can be inhibited by fulacimstat, albeit with 35-fold less potency than chymase (32). Faraday et al. (33) have shown that cathepsin G knockout mice have significantly prolonged bleeding time, suggesting that this enzyme plays a crucial role in modulating hemostasis *in vivo* (33). In contrast, consecutive administrations of fulacimstat did not alter bleeding time in healthy subjects or heart and renal failure patients, indicating no significant interference with cathepsin G activity (22 ; 23).

Finally, in this study, a thrombotic environment was artificially recreated by adding the exogenous chymase prior to halo coagulation, thus allowing the enzyme to be sequestered within the blood clot as previously observed in human DVT (15), prior to fibrinolysis. However, this assay confines the clot composition to the constituents found within the volume of blood used for clot formation, potentially differing from the heterogeneity typically observed in real thrombi, of venous or arterial origin.

In conclusion, the present studies support the hypothesis that human chymase is present and active in human clots and can contribute to reduced fibrinolysis by inactivating plasmin. The beneficial effects observed with chymase inhibitors in the resolution venous thrombi in animal models without affecting normal hemostasis may be translatable to human thrombus resolution. Further experiments are warranted to evaluate the usefulness of chymase inhibitors in potential clinical settings.

## Data Availability

The raw data supporting the conclusions of this article will be made available by the authors, without undue reservation.
