# Supplementary material for: Recombinant chymase inhibits fibrinolysis induced by endogenous plasmin in clotted human blood
Source: Front Immunol. 2025 Apr 17;16:1511990. doi: 10.3389/fimmu.2025.1511990 (PMC12043487; doi:10.3389/fimmu.2025.1511990)
Supplement: Supplementary file 1 [file DataSheet1.pdf]

Supplementary Material

1 Supplementary Data

1.1 Chymase-Dependent Fragmentation of Plasmin

Chymase specific cleavage sites on plasmin were identified using trypsin digestion method and identification of cleavage products by LC-MS/MS, as previously described (Lapointe *et al.*, 2023). Recombinant CMA-1 (rCMA-1) was produced as pro-forms in-house and activated using recombinant mouse cathepsin C (R&D Systems, Minneapolis, MN, USA) as described previously. The rCMA-1 was thawed and diluted to a concentration of 20 µg/mL in maturation buffer (50 mM MES, 0.1% w/v BSA, pH 5.5). Active murine cathepsin C was prepared at a concentration of 20 µg/mL in cathepsin C buffer (50 mM MES, 50 mM NaCl, 5 mM DTT, pH 5.5). Activation was achieved by combining equal volumes of recombinant chymase and cathepsin C, along with 50 µg/mL heparin, followed by a 1-hour incubation at room temperature. The chymase activation was then halted using 3 mM N-ethylmaleimide (NEM). The reaction mixture was diluted with assay buffer (20 mM Tris, 2 M KCl, 0.02% v/v Triton X-100, pH 9.0), adjusting the recombinant chymase concentration to 2 µg/mL. A 5-minute incubation was sufficient to completely stop the cathepsin C-dependent reaction.

To evaluate plasmin fragmentation, rCMA-1 (2 ng/µL) was pre-incubated with either vehicle or the chymase inhibitor fulacimstat (146 ng/µL) at 37°C for 20 minutes. This was followed by a 30-minute incubation at 37°C with human plasmin (20 ng/µL) (Innovative Research Inc., Novi, MI, USA). The reaction was terminated by adding formic acid (FA) to a final concentration of 4% v/v, and the samples were kept on ice until protein precipitation.

1.2 In-Solution Trypsin Digestion, Purification, and Desalting of Peptides

Proteins from the *in vitro* cleavage assays were precipitated by adding trichloroacetic acid (TCA) to a final concentration of 10% v/v, followed by a 30-minute incubation at -20°C. The precipitated proteins were collected by centrifugation at 10,000 ×g for 15 minutes. The resulting pellets were washed with cold 100% acetone, air-dried, and re-suspended in 50 µL of a solution containing 8 M urea and 10 mM HEPES-KOH (pH 7.4).

Protein reduction was performed by adding DTT (Thermo Fisher Scientific, Waltham, MA, USA) to a final concentration of 5 mM, heating at 95°C for 2 minutes, and incubating at room temperature for 30 minutes. Alkylation was carried out with iodoacetamide (Sigma-Aldrich, Saint-Louis, MO, USA) at a final concentration of 7.5 mM for 20 minutes in the dark. Urea concentration was then reduced to 2 M by adding 150 µL of 50 mM ammonium bicarbonate (NH4HCO3). Proteins were digested overnight at 30°C with 1 µg Pierce MS-grade trypsin (Thermo Fisher Scientific, Waltham, MA, USA). Digestion was stopped with trifluoroacetic acid (TFA) to a final concentration of 0.2% v/v.

Peptides were purified using Pierce C18 100-µL tips (Thermo Fisher Scientific, Waltham, MA, USA). The C18 tips were conditioned with 100 µL of 100% acetonitrile (ACN) three times, followed by equilibration with 0.1% v/v TFA buffer. Each sample was loaded onto the column by performing 10

successive cycles of aspiration and dispensing (100 µL per cycle). The procedure was repeated twice for complete sample loading. The column was washed three times with 100 µL of 0.1% v/v TFA buffer, and peptides were eluted into a low-binding microtube using 50% v/v ACN and 1% v/v FA, repeated three times for a total volume of 300 µL. Peptides were dried using a centrifugal evaporator at 65°C for approximately 60 minutes, then resuspended in 25 µL of 1% v/v FA. The concentration was measured at 205 nm using a NanoDrop spectrophotometer (Thermo Fisher Scientific), and samples were stored at -20°C until mass spectrometry analysis.

1.3 LC-MS/MS Analyses

Trypsin-digested peptides were analyzed using a Dionex Ultimate 3000 nanoHPLC system. A 10 µL sample (containing 2 µg of peptides) was loaded onto an Acclaim PepMap100 C18 column (0.3 mm id × 5 mm, Dionex Corporation) at 4 µL/min. Peptides were eluted onto an EasySpray PepMap C18 nano column (75 µm × 50 cm, Dionex Corporation) using a linear gradient of 5-35% solvent B (90% ACN with 0.1% FA) over 240 minutes, at a flow rate of 200 nL/min. An Orbitrap QExactive mass spectrometer (Thermo Fisher Scientific) coupled via an EasySpray source was used for detection, with spray voltage set to 2.0 kV and the column temperature maintained at 40°C.

Full-scan MS spectra (m/z 350-1600) were acquired with a resolution of 70,000, and the 10 most intense ions were selected for fragmentation by collision-induced dissociation (CID) at a normalized collision energy of 35% and resolution of 17,500. Data acquisition was controlled by Xcalibur software (Thermo Fisher Scientific).

1.4 Protein Identification by MaxQuant Analysis

Raw data were processed using MaxQuant (version 1.6.17.0) against the Uniprot human proteome databases (21/03/2020, 75,776 entries), including plasminogen entries (UniProtKB - P00747 for human). Settings included up to 4 missed cleavages, a minimum peptide length of 6, carbamidomethylation as a fixed modification, and variable modifications of methionine oxidation, N-terminal acetylation, and carbamylation. Trypsin and CMA1 were used as enzymes. Mass tolerances were 10 ppm for precursor ions and 20 ppm for fragment ions. FDR thresholds were set at 0.05 for PSM, protein, and site decoy fractions. Label-free quantification (LFQ) was performed with a minimum ratio count of 1. Proteins flagged as "Reverse," "Only.identified.by.site," or "Potential.contaminant" were excluded from the analysis.

2 Supplementary Figures

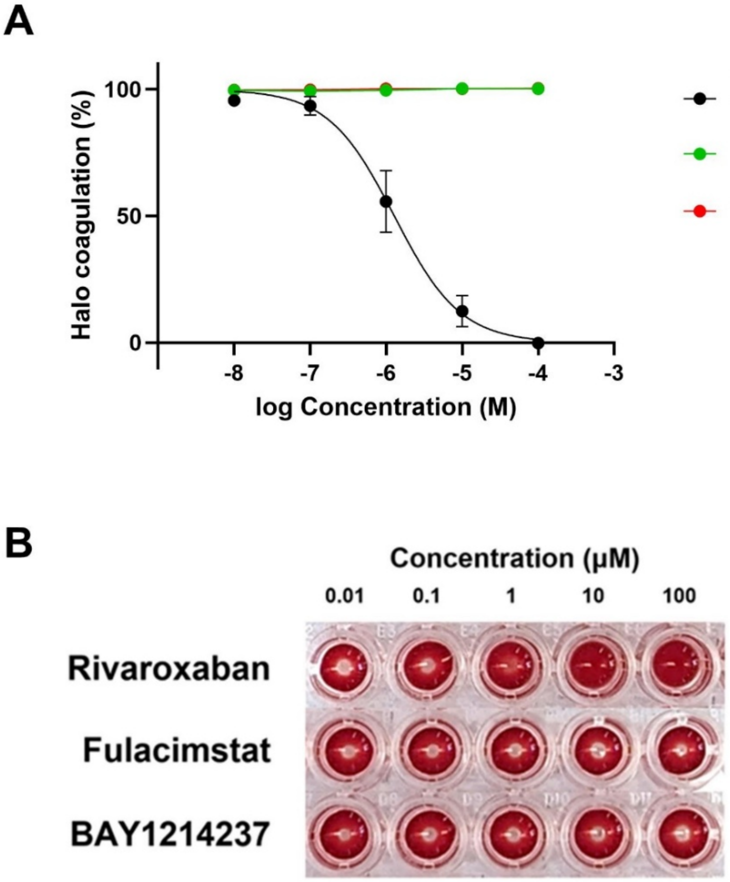

Supplementary Figure 1. (A) Measurement and **(B)** photos of blood halo coagulation in the presence of concentrations increasing levels of Rivaroxaban (Xarelto®), fulacimstat and BAY 1214237. Each point corresponds to the mean ± SEM. (n = 6-7).

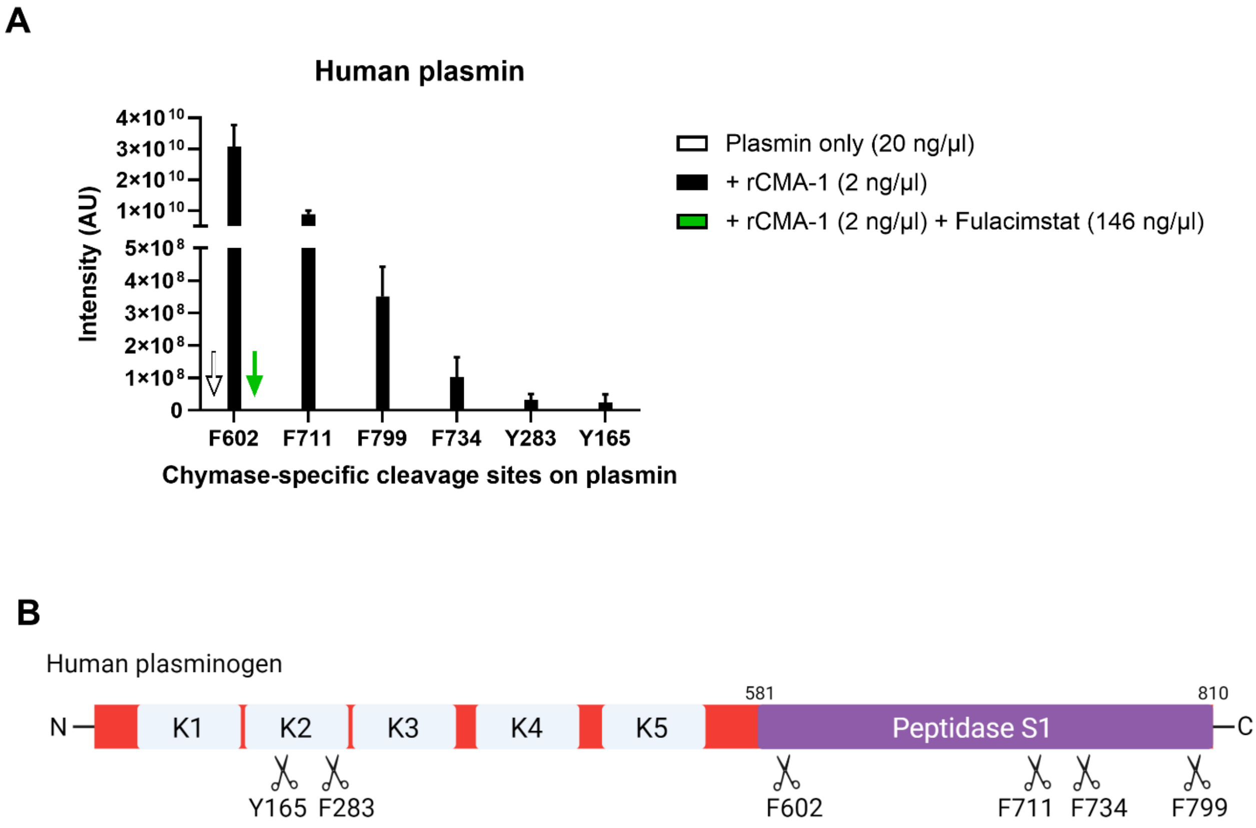

Supplementary Figure 2. Identification of enzymatic cleavage sites using trypsin digestion method and identification of cleavage products by LC-MS/MS. **(A)** Identification of proteolytic cleavage sites of rCMA-1 on purified human plasmin, sensitive to fulacimstat, based on intensity in arbitrary units (AU). Each bar corresponds to the mean ± SEM. (n = 4). **(B)** Schematic representation showing chymase-specific cleavage sites (black arrows) on the sequence of human plasminogen.
